# WIPO Re:Search: Catalyzing Public-Private Partnerships to Accelerate Tropical Disease Drug Discovery and Development

**DOI:** 10.3390/tropicalmed4010053

**Published:** 2019-03-26

**Authors:** Cathyryne K. Manner, Katy M. Graef, Jennifer Dent

**Affiliations:** BIO Ventures for Global Health, 2101 Fourth Avenue, Suite 1950, Seattle, WA 98121, USA; cmanner@bvgh.org (C.K.M.); kgraef@bvgh.org (K.M.G.)

**Keywords:** WIPO Re:Search, public-private partnerships, drug discovery, malaria, neglected tropical diseases, BIO Ventures for Global Health

## Abstract

Tropical diseases, including malaria and a group of infections termed neglected tropical diseases (NTDs), pose enormous threats to human health and wellbeing globally. In concert with efforts to broaden access to current treatments, it is also critical to expand research and development (R&D) of new drugs that address therapeutic gaps and concerns associated with existing medications, including emergence of resistance. Limited commercial incentives, particularly compared to products for diseases prevalent in high-income countries, have hindered many pharmaceutical companies from contributing their immense product development know-how and resources to tropical disease R&D. In this article we present WIPO Re:Search, an international initiative co-led by BIO Ventures for Global Health (BVGH) and the World Intellectual Property Organization (WIPO), as an innovative and impactful public-private partnership model that promotes cross-sector intellectual property sharing and R&D to accelerate tropical disease drug discovery and development. Importantly, WIPO Re:Search also drives progress toward the United Nations Sustainable Development Goals (SDGs). Through case studies, we illustrate how WIPO Re:Search empowers high-quality tropical disease drug discovery researchers from academic/non-profit organizations and small companies (including scientists in low- and middle-income countries) to leapfrog their R&D programs by accessing pharmaceutical industry resources that may not otherwise be available to them.

## 1. Introduction

Affecting more than one billion people globally, tropical diseases—including malaria and a group of infections termed neglected tropical diseases (NTDs) found primarily in hot, humid climates—pose enormous threats to human health and wellbeing, particularly in the world’s poorest populations. These infectious diseases cost developing economies billions of dollars annually [1]. The age-standardized disability-adjusted life-year (DALY) rate for malaria and NTDs combined was estimated to be 877.6 per 100,000 in 2017 [2]. Malaria alone was responsible for an estimated 219 million new cases and 435,000 deaths in 2017, predominantly in the World Health Organization (WHO) African Region. No significant reduction in malaria cases was seen between 2015 and 2017, and the decline in malaria mortality rates has slowed since 2015 [3].

The enormous burden of tropical diseases, as well as the imperative for collective action to eradicate or eliminate them, are evidenced by their inclusion in the United Nations (UN) Sustainable Development Goals (SDGs). SDG Target 3.3 aims to end the malaria and NTD epidemics by 2030 [4]. Increasing coverage of essential health services, including access to appropriate medicines, is critical for achieving this target [5]. Uniting to Combat NTDs—an international coalition of pharmaceutical companies, government entities, and academic and non-profit organizations—exemplifies the power of coordinated efforts by private- and public-sector stakeholders to scale up healthcare programs in endemic countries to battle tropical diseases. A key component of the initiative is drug donations from the pharmaceutical industry—totaling US$17 billion in commitments from 2012 to 2020—including Mectizan^®^ (ivermectin) for lymphatic filariasis and onchocerciasis from MSD (a trademark of Merck & Co., Inc., Kenilworth, NJ, USA), praziquantel (PZQ) for schistosomiasis from Merck KGaA, and Zithromax^®^ (azithromycin) for trachoma from Pfizer. Partnerships with governments, non-governmental organizations, and healthcare workers in the recipient countries help ensure that the donations reach those in greatest need. In 2016 alone, one-seventh of the world’s population, representing more than one billion people across the poorest countries, was treated for at least one NTD. The efforts of the Uniting to Combat NTDs coalition have also contributed to the elimination of lymphatic filariasis, onchocerciasis, and trachoma in multiple countries across the Americas, the Asia-Pacific region, and the Middle East [6].

In coordination with ongoing efforts to optimize and broaden access to current treatment regimens, expansion of research and development (R&D) of new tropical disease drugs is critical, and in alignment with SDG Target 3.B [4]. Despite substantial global interest in and commitment to tropical disease drug discovery and development, there remain significant unmet needs for new medications that address concerns associated with existing therapies, such as emergence of drug resistance [3,7,8]; lack of efficacy against infectious agents at all stages of their lifecycles [8]; lack of direct antimicrobial activity (i.e., only supportive care is available) [9]; and toxicity [10].

The reasons why such medical needs remain unmet are complex but include comparatively low R&D investment [11]—particularly for translational research [12]—and research intensity [13] for certain tropical diseases. Limited commercial incentives—particularly compared to products for diseases prevalent in high-income countries—have hindered many multinational pharmaceutical companies from bringing their immense product development expertise and resources to bear in the area of tropical diseases [12,14,15]. 

To address these issues, innovative and efficient models are needed that leverage the respective strengths of the public and private sectors to translate novel ideas into new tropical disease drugs in a coordinated, cost-effective, and mutually beneficial manner. In this article we present the WIPO Re:Search consortium—a global initiative co-led by BIO Ventures for Global Health (BVGH) and the World Intellectual Property Organization (WIPO)—as one such innovative and impactful public-private partnership model. As illustrated by the case studies herein, WIPO Re:Search empowers high-quality researchers from academic/non-profit organizations and small companies (including scientists in low- and middle-income countries [LMICs]) to leapfrog their tropical disease drug R&D programs by accessing pharmaceutical company resources that may not otherwise be available to them. The Consortium catalyzes cross-sector drug discovery and development across a broad array of tropical diseases, including conditions outside the current scope of product development partnerships (PDPs) such as Drugs for Neglected Diseases *initiative* (DND*i*) and Medicines for Malaria Venture (MMV). WIPO Re:Search also stimulates drug R&D focused on molecular entities that may fall outside of current PDP portfolios but that, given sufficient validation and interest, could potentially be incorporated into their pipelines. As such, WIPO Re:Search augments the important work of PDPs. 

## 2. WIPO Re:Search: Advancing Product R&D for Tropical Diseases and Other Diseases of Poverty Through a Novel Public-Private Partnership Approach

WIPO Re:Search, established in 2011, accelerates drug, vaccine, and diagnostic R&D for diseases of poverty—defined here as tropical diseases and other conditions that disproportionately affect LMICs (Table 1)—by bringing together the scientific expertise and creative thinking of academic and non-profit investigators, the firsthand disease knowledge of researchers in endemic countries, and the material assets and product development experience of global pharmaceutical companies. 

WIPO Re:Search catalyzes the royalty-free sharing of intellectual property (IP) assets such as compounds, data, clinical samples, technology, and expertise among Members to advance R&D for diseases of poverty. All data and IP generated through WIPO Re:Search R&D collaborations belong to the collaborators, not to BVGH or WIPO. The Consortium promotes access to any resulting products in the neediest populations by requiring IP contributors to agree, as a condition of membership and collaboration, to grant royalty-free licenses for product use and sale in all least developed countries (LDCs) and consider in good faith product access for all developing countries [16]. 

WIPO Re:Search membership currently spans over 140 organizations across over 40 countries and six continents, including eight pharmaceutical companies (Eisai Co., Ltd.; GlaxoSmithKline; Johnson & Johnson; Merck KGaA; MSD; Novartis; Pfizer; and Takeda Pharmaceutical Company, Ltd.); eight PDPs (DND*i*; FIND; GALVmed; International Vaccine Institute; MMV; PATH; Sabin Vaccine Institute; and Texas Children’s Hospital Center for Vaccine Development); and more than 120 academic and non-profit entities (including government organizations) [17]. While company Members pay an annual sponsorship fee to secure the benefits articulated below, WIPO Re:Search membership is free and open to all other interested organizations regardless of geography.

WIPO Re:Search membership delivers myriad benefits that drive robust cross-sector engagement (Table 2), including furtherance of business and corporate citizenship goals, advancement of R&D programs, enhanced international visibility, and scientific recognition. For entities and researchers in LMICs, WIPO Re:Search engagement also provides access to R&D- and career-enhancing resources not available in their home countries [18,19]. Such access bolsters the capacities of individual LMIC scientists, as well as entire institutions, to participate in and contribute to innovative research on diseases of poverty, in alignment with SDG 9 [4]. 

**Figure 1:**
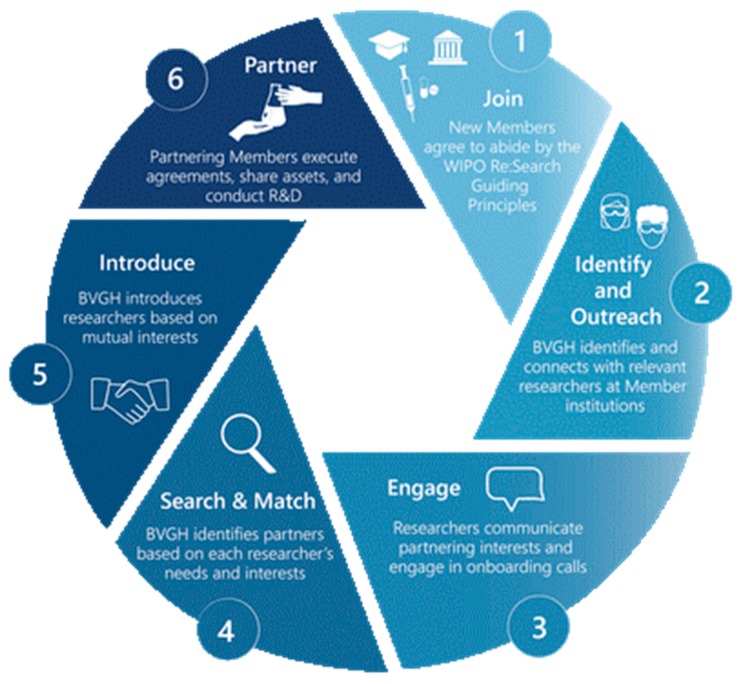
BVGH’s partnering process.

BVGH manages WIPO Re:Search collaboration development activities as the Partnership Hub Administrator, and has developed and implemented a unique process to connect Members for mutually beneficial R&D collaborations (Figure 1). BVGH identifies investigators and companies with critical capabilities, synergistic expertise, and complementary priorities and needs, and then introduces those parties to determine if there is reciprocal interest in collaborating. If so, BVGH facilitates communications between partners to align on milestones and agree on timelines and responsibilities. Once legal agreements are in place between the participating entities, BVGH provides alliance management support to help ensure successful outcomes. Depending on the specific needs of the collaboration, such support includes coordination of regular update calls, recruitment of additional partners with needed expertise, and leveraging of BVGH FundFinder to help collaborators identify relevant and high-value award opportunities. BVGH FundFinder—built in a sortable database and housed in a highly optimized format—is a unique, comprehensive source for funding opportunities, from over 245 public- and private-sector entities, relevant to diseases of poverty. The most recent issue of BVGH FundFinder included 269 open and upcoming funding opportunities totaling upwards of US$249 million.

BVGH’s matchmaking and partnering approach prioritizes projects (termed targeted collaborations) that address the greatest health burdens and gaps for diseases of poverty, as identified by analyses of product development pipelines and publications by leading experts. As targeted collaborations align with the priorities and interests of major global health funders and other stakeholders, BVGH’s approach positions projects to attract the financing and partners needed to transition innovation into impactful products. The criteria for targeted collaborations are listed in Table 3; projects do not need to satisfy all criteria to be considered targeted.

Over 140 WIPO Re:Search collaborations have been established to date, two-thirds of which are drug R&D projects [23]. The preponderance of such projects reflects researchers’ strong interest in repurposing pharmaceutical company compounds (primarily molecules developed for other indications) to efficiently accelerate drug development for diseases of poverty in which homologous molecular pathways are disrupted [15]. In addition to compounds, many industry/non-profit collaborations involve sharing of corporate validation data, technology platforms, and product development expertise to enable project success. Companies voluntarily collaborate and share their assets to contribute to R&D for diseases of poverty and save lives around the world. The WIPO Re:Search model provides companies with a wide array of opportunities to do so in a manner that maximizes company contribution to collaborations and resulting product impacts. For example, BVGH vets potential partners to confirm their mutual interest in and readiness for collaborations with multinational pharmaceutical companies; coordinates collaborator interactions across potential barriers of time zones and cultural differences; provides high-quality alliance management services; and, as needed, assists in addressing the unique challenges associated with conducting drug R&D in low-resource settings.

## 3. Case Studies: Leveraging Pharmaceutical Company Assets to Drive Tropical Disease R&D at Academic/Non-Profit Entities and Smaller Companies

Over 70 WIPO Re:Search collaborations, more than 30 of which are currently active, are pursuing drug discovery for tropical diseases. The majority of such collaborations involve a for-profit partner (typically a large pharmaceutical company) sharing IP with an academic/non-profit organization or a smaller company to propel drug R&D, including the following projects: 

### 3.1. Malaria

WHO has identified resistance to antimalarial drugs, particularly artemisinin and the partner medications that are the mainstay of treatment for uncomplicated *Plasmodium falciparum* malaria, as a major threat to malaria control. Such resistance has already been reported in the Greater Mekong subregion of Southeast Asia [3], underscoring the need for new antimalarial drugs with alternate mechanisms of action.

As part of its longstanding commitment to drug discovery for diseases of the developing world, MSD was interested in finding collaborators for an R&D program focused on evaluating its collection of aspartyl protease inhibitors for the treatment of malaria. With assistance from BVGH, MSD identified Professor Alan Cowman of the Walter and Eliza Hall Institute of Medical Research (WEHI) in Australia—who had previously validated the aspartyl protease plasmepsin V as an antimalarial drug target with essential roles in protein export and parasite survival in human erythrocytes [24,25,26]—as the best partner. Due to MSD’s prior experience with WIPO Re:Search, the company requested that BVGH catalyze and foster the collaboration through the Consortium. Professor Cowman and his colleagues Dr. Justin Boddey and Dr. Brad Sleebs screened MSD’s compounds in their high-throughput assays and identified hits, for which MSD is contributing its drug-hunting expertise to optimize potency, pharmacokinetics, and selectivity. After some initial encouraging results, BVGH advised the team to seek external funding via BVGH FundFinder to further advance the work. A funding opportunity from the Wellcome Trust, aimed at stimulating partnerships between for-profit and non-profit entities, was identified. MSD and WEHI jointly submitted a proposal and received an initial grant in 2015 and a second award in 2018, totaling more than US$3.5 million [27].

Professor Cowman and Dr. Sleebs are also working with Johnson & Johnson, in alignment with the company’s mandate to collaborate broadly in global health R&D, to develop drugs targeting critical *Plasmodium* pathways. BVGH facilitated this collaboration upon learning of the WEHI team’s interest in screening Johnson & Johnson’s Jump-stARter library, a set of 80,000 high-quality compounds representing significant structural and functional diversity. Hit-to-lead optimization is ongoing [28]. 

One of the most common, and often fatal, complications of *P. falciparum* infection is cerebral malaria, which causes seizures, coma [29], and, in survivors, long-term neurological and cognitive impairments [30]. Professor Alister Craig and his team at the Liverpool School of Tropical Medicine (LSTM) found that inhibition of the protease-activated receptor (PAR) 1 pathway might reduce brain swelling and mortality. After unsuccessfully reaching out to other companies to obtain PAR1 inhibitors, Professor Craig contacted BVGH, which connected him with Eisai Co., Ltd. through WIPO Re:Search. Seeking new opportunities to reposition its compounds to fight tropical diseases and potentially benefit millions of people worldwide, Eisai shared its PAR1 inhibitors (originally developed to treat coronary diseases) with the LSTM researchers. Based on promising initial results in an in vitro model of brain barrier function, Professor Craig was awarded a US$53,000 United Kingdom Medical Research Council Confidence in Concept grant to test additional Eisai PAR1 inhibitors using more complex models [31]. 

### 3.2. Schistosomiasis

Schistosomiasis affects an estimated 219 million people globally. PZQ is the only medication currently employed in mass drug administration (MDA) programs. However, as PZQ is not effective against immature schistosomes, repeated administration may be needed to ensure complete parasite clearance (as juvenile worms progress to maturity) and achieve acceptable cure rates. Reliance on a single agent also markedly increases the risk that resistance will develop [7,8]. There is a critical need to develop new therapeutics that target essential pathways that are not altered by PZQ. 

While at the University of California, San Francisco, Dr. Conor Caffrey validated *Schistosoma* 3-hydroxy-3-methylglutaryl coenzyme A reductase (*Sm*HMGR) as a novel drug target for schistosomiasis and demonstrated that commercial statin drugs, which inhibit the human homolog of *Sm*HMGR to treat hypercholesterolemia, have schistosomicidal activity [32]. Dr. Caffrey, now at the University of California, San Diego, subsequently launched a drug discovery program with the objective of translating these promising findings into new leads with high potency and selectivity against *Sm*HMGR. BVGH introduced Dr. Caffrey to research scientists at MSD, a pharmaceutical company with a history of statin discovery research, who provided statin analogs for his screening campaign. To inform future hit-to-lead optimization and medicinal chemistry efforts, BVGH also connected the collaborators to experts who are elucidating the crystal structure of *Sm*HMGR. Dr. Caffrey’s efforts on this project have been facilitated with competitively awarded funds from the National Institutes of Health [33].

### 3.3. Dengue

The global burden of dengue has been estimated at 390 million infections per year, although many cases are not reported or are misclassified. Severe dengue, known as dengue hemorrhagic fever or dengue shock syndrome, is a leading cause of hospitalization and death in Asia and Latin America. There is no specific treatment for dengue, although early detection and appropriate care by experienced healthcare providers can lower fatality rates below 1% [34]. 

BVGH facilitated a collaboration between two Member companies focused on blockade of platelet-activating factor receptor (PAFR) to treat dengue. Previous studies showed that treatment with the PAFR antagonist modipafant delayed and decreased lethality in mice inoculated with the dengue virus [35]. 60P Pharmaceuticals, a company that specializes in drug development for tropical diseases, was interested in repurposing modipafant for treating dengue fever. Modipafant was under development by Pfizer, but was discontinued. Pfizer disclosed modipafant’s Investigator’s Brochure to 60P under confidentiality. An Investigator’s Brochure is a compilation of the clinical and nonclinical data on the investigational product(s) that is relevant to the study of the investigational product(s) in human subjects [36].

### 3.4. Onchocerciasis

Onchocerciasis, or river blindness, affects an estimated 21 million people across the world, over one million of whom experience vision loss [37]. MDA campaigns with Mectizan have contributed to elimination of the disease in Colombia, Ecuador, Guatemala, and Mexico and increased coverage of individuals in many endemic communities [6]. However, while Mectizan is active against the first-stage larvae, or microfilariae (mf), of *Onchocerca volvulus*, it has limited efficacy against the adult worms (macrofilariae) that produce the mf [38]. Consequently, patients must take the medication at least annually to cover the full lifespan of the adult worm (up to 14 years) [39]. In addition, Mectizan also kills the mf of *Loa loa*—which is co-endemic with *O. volvulus* in many parts of Africa—leading to severe adverse events, including encephalopathy and death [40,41]. As a result, Mectizan MDA has been halted in certain African communities [42], and a new macrofilaricidal drug with no activity against *L. loa* mf is needed to protect those populations.

Professor Fidelis Cho-Ngwa at the University of Buea, Cameroon, has developed whole-parasite screening systems, in vitro and in vivo, to identify compounds that selectively kill both mf and adult *Onchocerca* spp. but not *L. loa* mf. To build on previous work demonstrating the potency of heat shock protein (Hsp) 90 inhibitors against *Brugia pahangi*, a microfilarial nematode similar to *O. volvulus*, Professor Cho-Ngwa connected with Merck KGaA through BVGH. With a strong interest in fostering collaborations between the global North and South to address IP barriers and accelerate infectious disease R&D, Merck KGaA shared 5600 compounds (including Hsp90 inhibitors and molecules targeting other critical parasitic pathways) with Professor Cho-Ngwa. 

Using BVGH FundFinder, BVGH identified the Wellcome Trust Pathfinder Award—which aims to stimulate partnerships between companies and academic scientists—as a potential funding source for this project. Professor Cho-Ngwa and Merck KGaA co-applied for the grant and received US$184,000, which supported initial screening of the compounds in Cameroon. A number of promising hits were identified. BVGH assisted the partners in identifying a medicinal chemistry expert to collaborate on hit-to-lead optimization, and they are co-preparing a second proposal to Wellcome Trust to support this work. As part of the ongoing partnership with Professor Cho-Ngwa, Merck KGaA will contribute expertise from departments such as Corporate Affairs, IP/Patents, Global Health, and Medicinal Chemistry to inform continued drug development [43].

## 4. Conclusions

WIPO Re:Search unites the for-profit sector and the international scientific community to streamline R&D and fuel the tropical disease product development pipeline in a cost- and resource-efficient manner. In addition to addressing unmet medical needs and the objectives of its Members, WIPO Re:Search aligns with and is responsive to the priorities and concerns of the larger global health community, including:

**SDGs.** By promoting product R&D to battle diseases of poverty, WIPO Re:Search is contributing to progress toward SDG 3, which aims to ensure healthy lives and promote wellbeing for all. In its capacity as an international public-private partnership, WIPO Re:Search is also addressing SDG 17, which focuses on increasing commitments to global partnerships and cooperation [4]. In particular, access to high-quality resources and know-how through multinational collaborations is helping to strengthen R&D enterprises in LMICs, in furtherance of SDG 9’s objectives of enhancing scientific research and innovation globally.

**Antimicrobial resistance (AMR).** WIPO Re:Search is establishing collaborations that target the growing epidemic of AMR. Projects are underway to develop novel drugs to outpace emerging antimalarial resistance and combat drug-resistant tuberculosis.

**The critical role of IP in driving innovation.** The WIPO Re:Search model focuses on the sharing of valuable IP—often from companies—to advance product R&D. As such, the Consortium demonstrates to diverse stakeholders that IP, which is often construed as a barrier to innovation and product development (particularly for diseases of poverty), can in fact promote such efforts. WIPO’s position as the UN agency responsible for global IP policy adds credibility and power to this messaging.

**Access to medicines.** Broad access to new products to prevent, detect, and treat diseases of poverty is at the core of WIPO Re:Search. All Consortium Members make public commitments to the WIPO Re:Search Guiding Principles, which include royalty-free license provisions for LDCs and good-faith consideration of product access in all LMICs [16].

The pharmaceutical industry plays an important role in accelerating the translation of basic research into product development for tropical diseases and other diseases of poverty, and yet many academic/non-profit researchers and small companies remain unaware of the partnership opportunities that are available to them through initiatives such as WIPO Re:Search. Scientists and companies interested in learning more about WIPO Re:Search membership or the Consortium in general are encouraged to email Cathyryne Manner (cmanner@bvgh.org) to schedule an introductory phone call to discuss their R&D needs and potential partnership opportunities within the WIPO Re:Search network. 

## Figures and Tables

**Table 1:** WIPO Re:Search fosters drug, vaccine, and diagnostic R&D for 21 diseases of poverty through IP sharing and targeted collaborations.

Parasitic Diseases	Bacterial Diseases	Viral Diseases	Other Conditions
Chagas disease	Buruli ulcer	Dengue	Podoconiosis
Cysticercosis	Leprosy	Rabies	Snakebite
Dracunculiasis	Trachoma		
Echinococcosis	Tuberculosis		
Foodborne trematodiases ^1^	Yaws		
Human African trypanosomiasis			
Leishmaniasis			
Lymphatic filariasis			
Malaria			
Onchocerciasis			
Schistosomiasis			
Soil-transmitted helminthiases			

^1^ Clonorchiasis, fascioliasis, opistorchiasis, and paragonimiasis.

**Table 2:** WIPO Re:Search engagement delivers significant value to companies, research institutions, and scientists interested in research and product development for diseases of poverty.

**Advancing Business and Corporate Social Responsibility Objectives**
Repurposing of valuable assets to stimulate R&D for diseases of poverty and improve health Opportunities to contribute to international dialogue and action on key global health issues (e.g., UN Sustainable Development Goals, antimicrobial resistance, IP as a driver of R&D innovation, access to medicines) Strengthening of Access to Medicine Index submissions through inclusion of WIPO Re:Search asset-sharing activities [20] (pp. 228, 244)
**Accelerating R&D Programs**
Customized R&D partnership development and alliance management services aligned with Members’ priorities and interests Access to technologies and expertise from other Members, saving valuable resources and time New perspectives on R&D for diseases of poverty through partnerships with endemic-country scientists Relationship building with UN agencies, governments, funders, researchers, and companies
**Expanding Global Brand Awareness and Visibility**
Public demonstration of commitment to sharing valuable IP to advance R&D for diseases of poverty Members and collaborations featured in WIPO Re:Search publications, presentations, and social media
**Increasing Scientific Recognition**
Peer-reviewed publications describing collaboration outcomes Increased eligibility and competitiveness for research funding
**Providing Additional Value for LMIC Organizations and Researchers**
Access to cutting-edge R&D resources not available in their home countries Opportunities to develop new skills through meaningful collaborations Career development (e.g., awards, promotions) enabled by publications and presentations on WIPO Re:Search activities

**Table 3:** Targeted collaboration criteria. BVGH’s partnering process emphasizes development of collaborations that meet critical healthcare needs in LMICs and are likely to result in products that reach patients.

#	Criterion	Rationale
1	Advanced stage of development (typically preclinical or later)	Advanced-stage collaborations have higher chances of successfully completing all phases of development.
2	Addresses an unmet medical need	Such projects have greater likelihood of receiving funding or being transferred to an appropriate partner, such as a PDP or company, for continued development.
3	Novel target or approach	Such collaborations have greater potential to lead to innovative products that address unmet needs.
4	Aligns with published target product profiles (TPPs) or target candidate profiles (TCPs) (e.g., [21,22])	TPPs and TCPs define experts’ standards and expected needs for on-the-ground product use. By aligning with TPP or TCP criteria, a product is more likely to meet experts’ standards and be accessible and acceptable for use in endemic regions.
5	Exemplifies the use of IP as an enabler of innovation, or demonstrates a company’s commitment to improving access to medicines	WIPO Re:Search has two core goals: to accelerate product development for diseases of poverty, and to demonstrate that IP is not a barrier to access to medicines. Projects that fulfill this criterion exemplify the latter goal.
